# Detection of anti-drug antibodies using a bridging ELISA compared with radioimmunoassay in adalimumab-treated rheumatoid arthritis patients with random drug levels

**DOI:** 10.1093/rheumatology/kew299

**Published:** 2016-08-25

**Authors:** Meghna Jani, John D. Isaacs, Ann W. Morgan, Anthony G. Wilson, Darren Plant, Kimme L. Hyrich, Hector Chinoy, Anne Barton

**Affiliations:** ^1^Arthritis Research UK Centre for Epidemiology, University of Manchester, Manchester Academic Health Science Centre, Manchester, UK; ^2^Arthritis Research UK Centre for Genetics and Genomics, Centre for Musculoskeletal Research, Institute of Inflammation and Repair, University of Manchester, Manchester Academic Health Science Centre, Manchester, UK; ^3^Musculoskeletal Research Group, Institute of Cellular Medicine, Newcastle University; ^4^National Institute of Health Research Newcastle Biomedical Research Centre, Newcastle upon Tyne, UK; ^5^Leeds Institute of Rheumatic and Musculoskeletal Medicine, University of Leeds, National Institute of Health Research Leeds Musculoskeletal Biomedical Research Unit, Leeds, UK; ^6^University College of Dublin School of Medicine and Medical Science, Dublin, Ireland; ^7^Arthritis Research UK Centre for Genetics and Genomics, Centre for Musculoskeletal Research, Institute of Inflammation and Repair, University of Manchester, Manchester Academic Health Science Centre, Manchester, UK; ^8^NIHR Manchester Musculoskeletal Biomedical Research Unit, Central Manchester University Hospitals NHS Foundation Trust, Manchester Academic Health Science Centre, University of Manchester, Manchester Academic Health Science Centre, Manchester, UK; ^9^Arthritis Research UK Centre for Epidemiology, Centre for Musculoskeletal Research, University of Manchester, University of Manchester, Manchester Academic Health Science Centre, Manchester, UK; ^10^National Institute of Health Research Manchester Musculoskeletal Biomedical Research Unit, University of Manchester, Manchester Academic Health Science Centre, Manchester, UK; ^11^Centre for Musculoskeletal Research, University of Manchester, Manchester Academic Health Science Centre, Manchester, UK; ^12^Arthritis Research UK Centre for Genetics and Genomics, Centre for Musculoskeletal Research, Institute of Inflammation and Repair, University of Manchester, Manchester Academic Health Science Centre, Manchester, UK

**Keywords:** immunogenicity, anti-drug antibodies, adalimumab, anti-TNF, TNF-inhibitors, ELISA, RIA, drug levels, therapeutic drug monitoring, human anti-human antibodies

## Abstract

**Objective.** To determine the concordance between RIA and bridging ELISA at detecting anti-drug antibodies (ADAbs) in the context of random adalimumab levels and investigate the additional clinical utility of detecting ADAbs in RA patients who test ADAb positive by RIA and negative by ELISA.

**Methods.** ADAb levels were determined using RIA and bridging ELISA in 63 adalimumab-treated RA patients (159 samples). Immunogenicity concordance was determined using receiver operating characteristic curves. To determine the additional clinical value provided by a positive RIA in the presence of negative ELISA, association between treatment response (ΔDAS28), adalimumab drug levels and ADAbs was evaluated longitudinally using generalized estimating equation.

**Results.** Of the 60 RIA^+^ samples (n = 31 patients), 19 (n = 10 patients) were also ELISA^+^, corresponding to 31.7% of samples. Area under the curve for detecting ADAbs using ELISA (compared with RIA) using receiver operating characteristic curves was 0.65 (95% CI: 0.59, 0.71); this increased to 0.91 (95% CI: 0.81, 0.99) if ADAbs were ⩾100 AU/ml using RIA. In RIA^+^/ELISA^−^ patients, adalimumab levels were associated with ΔDAS28 over 12 months [regression coefficient: 0.098 (95% CI: 0.043, 0.15), P < 0.0001] and while ADAbs were significantly associated with drug level, they were not directly associated with ΔDAS28 over 12 months [β coefficient: 0.00083 (95% CI: −0.0038 to 0.0054), P = 0.72].

**Conclusion.** ADAbs were detected using ELISA more frequently when present in high titres as measured by RIA. In RIA^+^/ELISA^−^ patients, only drug levels were significantly associated with treatment response. Although ADAbs were not independently associated with treatment response, they may be helpful in determining the aetiology of low drug levels.

Rheumatology key messages

- Compared with RIA, ELISAs demonstrated good specificity but poor sensitivity in RA patients with random drug level measurements.
- In RIA^+^/ELISA^−^ patients, only drug levels were significantly associated with treatment response.
- A sensitive anti-drug antibody assay is useful to determine the aetiology of low drug levels in RA.

## Introduction

In up to 40% of RA patients treated with an anti-TNF therapy, the drugs fail to control disease activity adequately due to primary or secondary inefficacy (loss of response). One explanation is immunogenicity leading to the development of anti-drug antibodies (ADAbs). ADAbs may reduce the efficacy of anti-TNF treatment, by competing for the cytokine-binding site (neutralizing antibodies) or by promoting more rapid drug clearance (non-neutralizing/binding antibodies), leading to sub-therapeutic drug levels. The majority (>97%) of ADAbs to adalimumab are neutralizing [1], thus immediately antagonizing TNF inhibition.

Bridging ELISAs and RIA have been most commonly utilized in clinical studies for ADAb detection [2, 3]. ELISAs have the advantages of low cost, high throughput and ease of automated testing in most clinical laboratories. However, the bridging ELISA may be less tolerant to the effects of free circulating drug as both Fab arms of the antibody need to be available for binding to the drug coated on the plate, as well as the biotinylated drug for detection. RIA uses protein A Sepharose to capture ADAb from the patient’s serum, followed by addition of radiolabelled drug, which binds to drug-specific antibodies. Fluid-phase RIA is not influenced by artefacts induced by solid-phase adsorption of proteins, and thus has the advantage over solid-phase ELISAs, better reflecting the situation *in vivo*. The RIA is more specific than the bridging ELISA, is less prone to drug interference and can also detect certain IgG subclasses, namely IgG1, IgG2 and IgG4 (which are functionally monovalent [only bound to ‘antigen’] and which have a greater potential for neutralization [4]). Radioisotopes, however, make RIA more complex to set up and expensive than ELISA, which limits widespread use.

We have previously demonstrated that ADAbs to monoclonal antibodies, as measured by RIA, lead to low drug levels and are important predictors of poor treatment response. This held true even in the presence of free drug and when assessed at random points in the treatment cycle (herein referred to as random drug levels, which are not necessarily collected before the patient is due the next dose) [5]. A combination of ADAbs to adalimumab and low drug levels at 3 months generated an area under the receiver operating characteristic (ROC) curve (AUC) of 0.71 (95% CI: 0.57, 0.85) for lack of EULAR response at 12 months, suggesting good predictive utility for clinical practice. To implement immunogenicity testing successfully in a clinical setting, a less expensive and simpler test, such as an ELISA, would be preferable. In a practical clinical setting, trough levels in blood samples taken immediately prior to next drug dosing, although maximally informative, are difficult to obtain. While our previous work demonstrated the utility of random samples in the context of RIA ADA testing, the clinical value of ELISA in this setting is unknown [5]. Our aims were to determine the concordance between RIA and a commercially available ELISA in adalimumab-treated RA patients, in the context of random blood samples, and evaluate the additional clinical utility of ADAbs that are detectable by RIA but not by ELISA.

## Methods

### Study population

Patients were recruited to a prospective observational cohort study, the Biologics in Rheumatoid Arthritis Genetics and Genomics Study Syndicate [6], between November 2008 and March 2013. From the total cohort, patients were selected according to the following inclusion criteria: RA according to the revised ACR 1987 criteria [7], active disease indicated by a DAS28 ⩾ 5.1 despite earlier treatment with at least two DMARDs including MTX; patients of Caucasian descent; about to be initiated on adalimumab (40 mg every fortnight). At baseline and following initiation of therapy, patients had serum samples collected with disease activity measured at 3, 6 and 12 months. Treatment response was determined using change in DAS28CRP from baseline (ΔDAS28, defined as baseline DASCRP score-time point 3, 6 and 12 months DASCRP score). An improvement with treatment therefore would lead to a positive value ΔDAS28CRP. EULAR response criteria were calculated for descriptive purposes [8]. All participating patients provided written informed consent and the study was approved by a multicentre ethics committee (COREC 04/Q1403/37).

### Measurement of ADAbs and drug levels

All adalimumab samples (n = 414) in 160 patients were tested for ADAbs using RIA (Sanquin) and drug levels as previously described [5, 9]. Drug levels were measured using sandwich ELISAs manufactured by Progenika Biopharma, Derio, Spain. Additionally in 159 samples in 63 patients, which included all ADAb positive patients as well as a random selection of negative ADAb patients, serum ADAbs were measured using a commercially available bridging ELISA (Progenika Biopharma). Measurement of ADAbs and drug levels was performed in-house according to the manufacturer’s instructions. Patients were deemed to be ADAb positive by ELISA if levels detected were ⩾3.5 AU/ml and ADAb positive by RIA if levels were >12 AU/ml, as per the manufacturer.

### Statistics

**Fig. 1:**
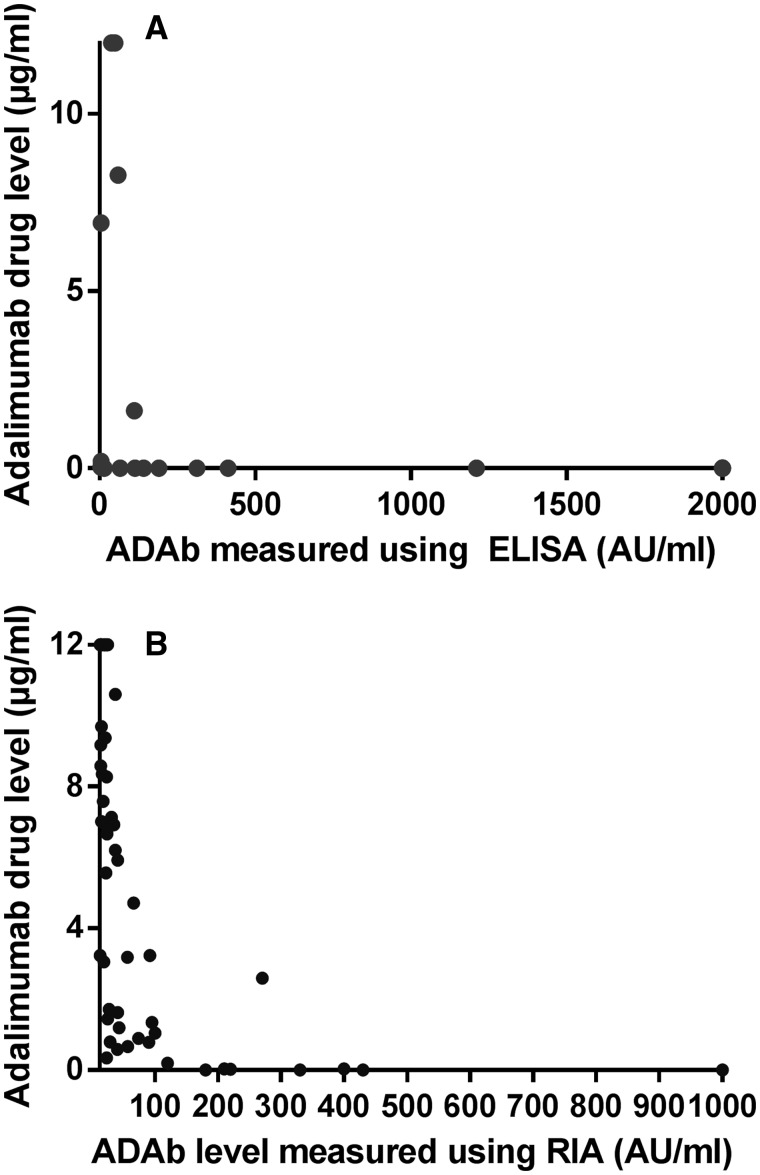
Drug tolerance of bridging ELISA (A) and RIA (B) ELISA detected ADAb levels demonstrated up to 2000 AU/ml; RIA detected ADAb levels demonstrated up to 1000 AU/ml.

Between group comparisons were evaluated using Mann–Whitney U (Wilcoxon) statistics and chi-squared tests as appropriate. Non-parametric Spearman’s correlations were determined between adalimumab drug level and ADAb using both RIA and ELISA, as well as ADAbs detected using both techniques. Kappa coefficient values were calculated for comparisons between both techniques. Area under the ROC curve (AUC) was determined to test the sensitivity of ELISA at detecting ADAbs when compared with detection using RIA. The generalized estimating equation (GEE) model with an identity link for longitudinal continuous outcomes was used to test the association between treatment response, drug and ADAb levels in patients who had ADAbs detected using RIA but not ELISA, to assess the value of detecting additional RIA positive samples. Statistical analyses were performed using STATA for Windows version 13.0 (Stata Corp., College Station, TX, USA) and Graph Pad Prism 6.04 for generation of Figure 1.

## Results

One hundred and fifty-nine samples in 63 patients were tested for ADAbs to adalimumab using both techniques. Of the 60 samples that were positive using RIA (n = 31 patients) [5], 31.7% tested positive using ELISA (19 samples in 10 patients). In patients in whom ADAbs were detected using an ELISA, titres continued to increase for the following 3 months (Table 1). Spearman’s correlation with adalimumab drug levels was as follows: ELISA r_s_ −0.45 (P < 0.001); RIA r_s_ −0.51 (P < 0.001). This demonstrated an inverse association between drug and ADAb levels using both techniques. Overall correlation between ADAbs detected by ELISA and RIA was moderate, but much stronger when high titre ADAbs were detected using RIA at levels >100 AU/ml (Table 1). Similarly the AUC for detecting ADAbs by performing an ELISA (compared with RIA) using ROC curves was 0.65 (95% CI: 0.59, 0.71); this increased to an AUC of 0.91 (95% CI: 0.81, 0.99) in samples in which ADAbs were detected at concentrations of ⩾100 AU/ml using RIA [18 samples with ADAbs ⩾100 AU/ml (range 100–111 000)]. Of the 21 samples testing positive using ELISA, the majority (n = 15; 71.4%) were in samples with ADAb titres of ⩾100 AU/ml using RIA. Kappa coefficient, sensitivity, specificity, and positive and negative predictive values are detailed in Table 1.

**Table 1:** Patient characteristics and concordance between RIA or ELISA for anti-drug antibody testing

Adalimumab drug levels in patients stratified by anti-drug antibody status using RIA and ELISA						
	Time point					
Variable	3 months		6 months		12 months	
	Median (IQR)	P-value^a^	Median (IQR)	P-value	Median (IQR)	P-value^a^
Adalimumab drug level if ADAb negative using RIA, µg/ml	12.0 (11.4–12.0)	<0.001	12.0 (11.9–12)	<0.001	12 (7.9–12)	<0.001
Adalimumab drug level if ADAb positive using RIA, µg/ml	4.6 (0.9–8.2)		2.1 (0–8.7)		1.7 (0–6.8)	
ADAb level using RIA, AU/ml^b^	37 (23–95)	—	48.5 (18–200)	—	25 (21–2,800)	—
Adalimumab drug level if ADAb negative using ELISA, µg/ml	9.9 (5.2–12)	0.08	11.0 (4.7–12)	<0.001	4.6 (1.8– 11.3)	<0.001
Adalimumab drug level if ADAb positive using ELISA, µg/ml	1.6 (0.2–8.2)		0 (0–0.03)		0 (0–0)	
ADAb level using ELISA, AU/ml	59.1 (49.3–111.5)	—	141.6 (38.9–312.2)	—	2000 (14.8–2000)	—

Patient characteristics in those who are ADAb positive using both test *vs* patients who are ADAb positive using RIA and ADAb negative using ELISA			
Variable	ADAb positive using both tests (RIA and ELISA)^b^	ADAb positive using RIA and ADAb negative using ELISA^c^	P-value^a^
ADAb level (using RIA), median (IQR), AU/ml	430 (120–4000)	25.0 (18.0–49.5)	<0.001
Adalimumab drug level, median (IQR), µg/ml	0 (0–0.20)	6.1 (1.4– 9.5)	<0.001
MTX use, patients, n (%)	5 (50)	12 (48)	0.98
MTX dose, median (IQR), mg/week	15 (7.5–22.5)	15 (10–22.5)	0.81
Disease duration median (IQR), years	12 (6.2–18.3)	14 (7.8–20.1)	0.63

GEE analysis in patients who were ADAb negative using ELISA and ADAb positive using RIA^c^		
Variable	Regression coefficient (95% confidence intervals)	P-value
Association between adalimumab drug levels and ADAbs using GEE	−3.15 (−4.41, − 1.88)	<0.0001
Association between treatment response (ΔDAS28) and adalimumab drug level	0.098 (0.043, 0.15)	<0.0001
Association between treatment response (ΔDAS28) and ADAb level	0.00083 (−0.0038, 0.0054)	0.72

Concordance between RIA and ELISA	Value (95% Confidence Intervals)	P-value
Kappa coefficient (95% CI)	0.35 (0.21, 0.48)	<0.001
Spearman’s correlation coefficient (all samples)	0.54 (0.42, 0.64)	<0.001
Spearman’s correlation coefficient (high titre ADAbs detected using RIA, ≥100 AU/ml)	0.86 (0.66, 0.95)	<0.001
AUC for detecting ADAbs using ELISA (all samples)	0.65 (0.59, 0.71)	
AUC for detecting ADAbs using ELISA high titre ADAbs detected using RIA, ≥100 AU/ml)	0.91 (0.81, 0.99)	
Sensitivity of ELISA (95% CI)	32.2% (20.6, 45.6)	
Specificity of ELISA (95% CI)	98% (93.0, 99.8)	
Positive predictive value	90.5% (69.6, 98.8)	
Negative predictive value	71.0% (62.7, 78.4)	

Adalimumab drug levels could be detected up to a maximal concentration of 12 µg/ml. MTX dose and disease duration are the described characteristics in the table as these were the two factors associated with ADAb formation in our cohort [5].

^a^P-value represents the significance of differences between groups using chi-squared tests for categorical outcomes and Wilcoxon rank sum tests for continuous variables.

^b^10 patients, 19 samples.

^c^25 patients, 40 samples. ADAb: anti-drug antibodies; AU: arbitrary units; AUC: area under the curve; GEE: generalized estimating equation.

Adalimumab levels were significantly different in patients who had ADAbs compared with patients who did not, using either method at 6 and 12 months (Table 1). Five patients had positive ADAbs using ELISA at 3 months (compared with 19 patients using RIA) (Table 1). Only four samples yielded both circulating drug and ADAbs by ELISA whereas the majority of samples that tested positive for ADAbs by RIA also demonstrated circulating drug (Fig. 1). High titre ADAbs (>100 AU/ml) were associated with absent drug levels using either technique.

Forty samples (in 25 individual patients) had ADAbs detected using RIA, but not using ELISA, the characteristics of which are shown in Table 1. To assess the effect of ADAbs detected using RIA on drug level longitudinally over 12 months, our previous work demonstrated a strong inverse association between adalimumab drug levels and ADAb status using the GEE: regression coefficient (RC) −4.77 [95% CI: −6.39 to −3.15], P < 0.0001 [5]. To quantify how much of the point estimate was attributed to these 40 samples, which would have been missed using ELISA, we performed an additional GEE model using only these patients (Table 1). This shows that the RC is lower (fewer samples and lower ADAb titre in this model) but continues to be highly significant [RC −3.70 (95% CI: −5.01 to −2.32), P < 0.0001]. Adalimumab drug levels in these samples continued to be significantly associated with ΔDAS28 over the course of 12 months. However, in the univariate analysis, ADAb level was no longer associated directly with treatment response (Table 1). Therefore the use of circulating drug levels alone provides a useful indicator of future treatment effect but detection of ADAb positivity in non-trough blood samples using RIA, otherwise missed using ELISA, provides additional value to the clinician interpreting the aetiology of a low adalimumab drug level.

Interestingly two samples tested positive using ELISA, but were negative using RIA (supplementary Fig. S1, available at *Rheumatology* Online). In one patient ADAb levels measured 14.8 AU/ml, with undetectable adalimumab levels, but the patient was found to have a good EULAR response at 12 months. It is possible that this patient (on MTX 10 mg/week) may have reached drug-free remission, no longer requiring an anti-TNF agent. In the second patient, ADAb levels of 49.3 AU/ml were detected at 3 months only, with an adalimumab level of >12 µg/ml (in association with a moderate EULAR response). These appear to be false-positive or transient antibodies, of no clinical significance.

## Discussion

Our study demonstrates, for the first time, utility of testing for ADAbs using ELISA in the context of random rather than trough drug levels, random levels being more practical to obtain in clinical practice. Of the two tests studied, ADAb detection using RIA was more sensitive in the presence of free drug compared with ELISA. Patients who had ADAbs detected using ELISA were more likely to have high titre ADAbs (>100 AU/ml) as detected by RIA. In patients in whom ADAbs were detected using RIA but not ELISA, ADAb levels failed to reach statistical significance independently in association with treatment response. However adalimumab levels continued to remain significantly associated with treatment response longitudinally across all time points and therefore were confirmed to be an important prognostic indicator.

Strengths of this study include the prospective serial sampling and well-characterized cohort of patients with treatment outcome measures. Previous studies that have tested for immunogenicity and concordance between tests have measured these in trough adalimumab samples [10, 11], but it is recognized that obtaining these in clinical practice is more challenging to perform and has practical implications for both service delivery and for the patients themselves. Although we compared ADAbs detected by ELISA with those detected by RIA (being the two more commonly performed tests in immunogenicity studies), currently there is no gold standard for measurement of ADAbs, detection of which may be influenced by a number of factors [12]. Newer, more drug tolerant assays continue to emerge that may be more suited for ADAb detection in the context of random drug levels, such as the pH-shift anti-idiotype antigen binding test, acid-dissociation RIA and temperature-shift RIA [13]. However, these tests are cumbersome and expensive to perform on a large clinical scale, are available in specialist centres only, and their utility in a clinical context is not yet known.

Our work highlights the importance of interpreting ADAb results in the context of simultaneously measured drug levels. The latter appears to be the more important of the two tests at predicting treatment response, especially in patients with lower ADAb titres. It is important to note that detection of ADAbs need not significantly influence treatment response if sufficient drug is still in circulation, which may explain results in RIA^+^/ELISA^−^ patients. While we acknowledge the limited power of our study, measurement of ADAbs using sensitive assays may provide valuable insight into the aetiology of low drug levels in adalimumab-treated patients. In a patient with a low circulating drug level, immunogenicity testing helps to determine causation, which in turn should optimize future management of the disease. For instance if ADAbs are detected in the context of a low drug level, switching to a less immunogenic drug could be beneficial [14] whereas switching to another mAb may trigger another immunogenic response and subsequent inefficacy [15]. These patterns may be missed when testing for ADAbs by ELISA in random samples and our previous work demonstrated that a low drug level may not always result from immunogenicity. An isolated low drug level (in the absence of detectable ADAbs) may be due to factors such as high BMI or poor adherence to therapy, both of which require different strategies compared with those for patients with detectable ADAbs [9].

In conclusion, when testing for immunogenicity at random points in the biologic treatment cycle, ELISA was less sensitive than RIA, with better concordance between the assays when ADAb titres were high (>100 AU/ml by RIA). Testing non-trough samples using ELISA can still demonstrate ADAbs but may be less clinically useful due to the high proportion of false-negative samples, most likely due to the poor tolerance of ELISA to free drug. Adalimumab drug level was the most important predictor of treatment response in patients who had ADAbs detected using RIA but not ELISA. However, a more drug tolerant assay such as RIA enables interpretation of the aetiology of low non-trough drug levels.

## Supplementary Material

Supplementary Data
