# Supplementary material for: Detection of anti-drug antibodies using a bridging ELISA compared with radioimmunoassay in adalimumab-treated rheumatoid arthritis patients with random drug levels
Source: Rheumatology (Oxford). 2016 Aug 25;55(11):2050–5. doi: 10.1093/rheumatology/kew299 (PMC5088626; doi:10.1093/rheumatology/kew299)
Supplement: Supplementary Data [file supp_55_11_2050__index.html]

Detection of anti-drug antibodies using a bridging ELISA compared with radioimmunoassay in adalimumab-treated rheumatoid arthritis patients with random drug levels — Supplementary Data 

# Detection of anti-drug antibodies using a bridging ELISA compared with radioimmunoassay in adalimumab-treated rheumatoid arthritis patients with random drug levels

## Supplementary Data

files

- Supplementary Data - docx file
