# Supplementary material for: Detection of anti-drug antibodies using a bridging ELISA compared with radioimmunoassay in adalimumab-treated rheumatoid arthritis patients with random drug levels
Source: Rheumatology (Oxford). 2016 Aug 25;55(11):2050–5. doi: 10.1093/rheumatology/kew299 (PMC5088626; doi:10.1093/rheumatology/kew299)
Supplement: Supplementary Data [file supp_kew299_rhe-15-1706-File004.docx]

**SUPPLEMENTARY DATA**

**Supplementary Figure S1: Concordance between RIA and ELISA for immunogenicity testing**

159 samples tested (in 63 patients)

62 samples tested +ve for ADAbs; 97 samples -ve for ADAbs

21 samples +ve for ADAbs using ELISA (n=12 patients)

60 samples +ve for ADAbs using RIA (n=31 patients)

2 samples (n=2 patients) +ve for ELISA only, not RIA

19 samples (n=10 patients) +ve for ADAbs using both ELISA and RIA

1 sample (n=1 patient) not tested for ADAbs using ELISA (haemolysed sample)

40 samples (n=25 patients) +ve for ADAbs using RIA but not ELISA

Abbreviations: ADAbs, anti-drug antibodies; ELISA, enzyme-linked immunosorbent assay; RIA, radioimmunoassay
